# Variable Release of Lipoteichoic Acid From *Staphylococcus aureus* Bloodstream Isolates Relates to Distinct Clinical Phenotypes, Strain Background, and Antibiotic Exposure

**DOI:** 10.3389/fmicb.2020.609280

**Published:** 2021-01-14

**Authors:** Marquerita Algorri, Peter Jorth, Annie Wong-Beringer

**Affiliations:** ^1^School of Pharmacy, University of Southern California, Los Angeles, CA, United States; ^2^Department of Pathology and Laboratory Medicine, Cedars-Sinai Medical Center, Los Angeles, CA, United States; ^3^Department of Medicine, Cedars-Sinai Medical Center, Los Angeles, CA, United States; ^4^Department of Biomedical Sciences, Cedars-Sinai Medical Center, Los Angeles, CA, United States

**Keywords:** antibiotics, immunomodulation, *Staphylococcus aureus* bacteremia, lipoteichoic acid, ceftaroline

## Abstract

**Background:**

*Staphylococcus aureus* is a leading cause of bacterial bloodstream infections. The heterogeneity in patient outcomes in *S. aureus* bacteremia (SAB) can be attributed in part to strain characteristics, which may influence host response to infection. We specifically examined the relationship between lipoteichoic acid (LTA) release from *S. aureus* and disease phenotype, strain background, and antibiotic exposure.

**Methods:**

Seven strains of *S. aureus* causing different clinical phenotypes of bacteremia and two reference strains (LAC USA 300 and Mu3) were analyzed for LTA release at baseline and following exposure to antibiotics from different pharmacologic classes (vancomycin, ceftaroline, and tedizolid). LTA release was quantified by LTA-specific ELISA. Whole genome sequencing was performed on the clinical strains and analyzed using open-source bioinformatics tools.

**Results:**

Lipoteichoic acid release varied by 4-fold amongst the clinical strains and appeared to be related to duration of bacteremia, independent of MLST type. Low LTA releasing strains were isolated from patients who had prolonged duration of bacteremia and died. Antibiotic-mediated differences in LTA release appeared to be associated with MLST type, as ST8 strains released maximal LTA in response to tedizolid while other non-ST8 strains demonstrated high LTA release with vancomycin. Genetic variations related to the LTA biosynthesis pathway were detected in all non-ST8 strains, though ST8 strains showed no variations despite demonstrating differential LTA release.

**Conclusion:**

Our findings provide the basis for future studies to evaluate the relationship between LTA release-mediated host immune response and clinical outcomes as well as the potential for antibiotic modulation of LTA release as a therapeutic strategy and deserve confirmation with larger number of strains with known clinical phenotypes.

## Introduction

*Staphylococcus aureus* is a leading cause of bloodstream infections and bacterial sepsis associated with significant morbidity and mortality (Naber, 2009; Liu et al., 2011; Mayr et al., 2014). Previous studies have demonstrated notable heterogeneity in clinical presentation and outcomes in patients with *S. aureus* bloodstream infections (SAB) (Naber, 2009; van Hal et al., 2012). While some patients respond quickly to antimicrobial therapy, one in three patients experience bacterial persistence, defined by prolonged positive blood cultures beyond 72 h of initiation of appropriate antibiotic therapy (Chong et al., 2013; Minejima et al., 2016; Guimaraes et al., 2019). Persistence is associated with metastatic complications and increased mortality (Hawkins et al., 2007; Rose et al., 2012; van Hal et al., 2012; Minejima et al., 2016) and our group has shown recently that each day of sustained growth of bacteria in the blood increases the risk of mortality by 16% (Minejima et al., 2020). The observed heterogeneity in clinical outcomes can be attributed, in part, to differential expression of virulence factors and resistance mechanisms across *S. aureus* strains (Minejima et al., 2016; Cyr et al., 2017; Pérez-Montarelo et al., 2018). Importantly, strain-specific factors within genetically diverse *S. aureus* strains have been shown to contribute to the development of immune dysregulation and subsequently, SAB persistence (Lotz et al., 2004; Hattar et al., 2006; Wang et al., 2012).

Clinical *S. aureus* strains commonly contain an array of diverse virulence factors that aid its survival within the host, including factors that promote immune evasion, biofilm formation, enhanced growth in serum, and toxin-mediated lysis of neutrophils and platelets (Oogai et al., 2011; Powers and Wardenburg, 2014). The repertoire of genes encoding virulence factors is uniquely associated with strain sequence type (ST) or clonal complex (CC), with CC8, CC5, and CC30 comprising the largest clades and most common causes of bacteremia (McCarthy and Lindsay, 2010; Cheung et al., 2014; Dabul and Camargo, 2014; Pérez-Montarelo et al., 2017). The bacterial cell wall contains many immunomodulatory components that are recognizable by the host. Early during infection, the innate immune response focuses upon targeting and recognizing bacterial cell wall components including peptidoglycan and lipoteichoic acid (LTA) (von Aulock et al., 2003; Fournier, 2013). Cell wall-related factors such as LTA are recognized expediently by neutrophils, the first-responder innate immune cell type (Spaulding et al., 2013). LTA is of particular interest due to its presence in all *S. aureus* strains and its role in stimulating pro-inflammatory cytokine response, activating neutrophils, delaying apoptosis, and modulating phagocytic response, all of which contribute to its potential role in the development of sepsis and severe infection by altering pro-inflammatory responses (Lotz et al., 2004; Fournier and Philpott, 2005; Seo et al., 2008; Thammavongsa et al., 2015). Of further interest, certain antibiotics such as cell-wall active agents have been reported to increase LTA release from *S. aureus* (van Langevelde et al., 1998; Lotz et al., 2006). In this way, antibiotics may indirectly influence the outcome of infection by modulating release of LTA, thereby activating host immune cells to produce pro-inflammatory cytokines.

Given the ubiquitous presence of LTA across *S. aureus* strains and its effect on the host immune response, we hypothesized that clinical strains may demonstrate differential release of LTA, which may affect clinical outcomes in patients with SAB. In this preliminary study, we selected *S. aureus* bloodstream isolates obtained from patients with distinct clinical phenotypes representative of persistent vs. resolving bacteremia and patient survival vs. death to: (1) examine strain-specific LTA release, (2) compare LTA release upon exposure to anti-staphylococcal antibiotics from different pharmacologic classes (vancomycin, tedizolid, ceftaroline), and (3) perform whole genome sequencing analyses to identify genetic differences in our study strains focusing on genes that could affect synthesis, release, and post-translational modification of LTA.

## Materials and Methods

### Selection of Bacterial Isolates

Study strains were collected from adult patients hospitalized for *S. aureus* bloodstream infection enrolled in a multicenter prospective observational study approved by the Institutional Review Board of the University of Southern California as published previously (Minejima et al., 2016, 2020). Selection of seven *S. aureus* strains (four MRSA, three MSSA) for this study was based upon distinct microbial and clinical phenotypes intended to represent a wide variety of clinical presentations of bloodstream infection. Relevant patient and microbial characteristics that were previously published are presented here for the selected study strains in Table 1.

**TABLE 1:** Selected *S. aureus* bloodstream isolates with associated clinical and microbial characteristics.

**Strain ID**	**Patient characteristics**	**Duration of bacteremia (Days)**	**Source of bacteremia**	**Outcome**	**Resistance type**	**MLST**	**spa type**	**SCCmec type**
HH35	70 y/o female	17	Dialysis catheter	Persistent, Died	MRSA	ST97	t267	IV (2B)
LA82	56 y/o male	17	Wound	Persistent, Survived	MRSA	ST8	t955	IV (2B)
HH70	60 y/o female	11	Unknown	Persistent, Died	MSSA	ST72	t148	I (1B)
HH92	66 y/o male	7	Osteomyelitis, non-spinal abscess	Persistent, Survived	MSSA	ST30	t338	I (1B)
HH131	47 y/o male	7	IV line	Persistent, Survived	MRSA	ST8	t008	IV (2B)
LA164	34 y/o female	1	Unknown	Resolving, Survived	MSSA	ST188	t189	V (5C2)
HH37	66 y/o male	1	IV line, surgical wound	Resolving, Survived	MRSA	ST5	t242	II (2A)

*All strains were isolated from blood obtained from culture-positive patients hospitalized in 2 United States medical centers. Persistence was defined by continued positive blood cultures following at least 72 h of appropriate antibiotic therapy, whereas resolving infections were culture negative less than 72 h of receipt of antibiotics. Survival was considered at 30 days of admission. Whole genome sequencing of bacterial strains was performed using Illumina MiSeq to determine MLST types and Protein A (spa) types.*

Factors that were considered for selection included: persistent versus resolving infection; patient 30-day mortality or survival; and methicillin-resistant *S. aureus* (MRSA) versus methicillin-susceptible *S. aureus* (MSSA) as well as strain background. Bacterial persistence was defined by positive blood cultures 72 h or more following initiation of appropriate antibiotic therapy, whereas resolving infections were characterized by negative blood cultures in less than 72 h. The isolates selected originate from common sources of bacteremia including skin and soft tissue infection, wound infection, abscess, catheter-related or unknown source.

Two well-characterized MRSA clinical reference strains, Mu3 and LAC USA 300, were included as control strains for comparison of LTA release and genomic variations. Mu3, a vancomycin heteroresistant sequence type (ST) five MRSA strain with a thickened cell wall, was isolated from a patient in Japan in 1997 (Hiramatsu et al., 1997; Cui et al., 2000). LAC USA 300 is an ST8 community-associated MRSA strain that is among the most common clonal strains in the United States, notable for its virulence potential and spread (Diekema et al., 2014; Kong et al., 2016). Both Mu3 and LAC strains are commonly used in experimental *in vitro* and *in vivo* models and their whole genomes have been sequenced, assembled, and annotated, and are accessible on public sequence databases such as GenBank.

### Generation of Bacterial Supernatants

Seven clinical strains (four MRSA, three MSSA) and two well-characterized MRSA reference strains (USA300, Mu3) were analyzed for LTA release in bacterial culture medium, with and without the presence of antibiotics. *S. aureus* strains were incubated in tryptic soy broth (TSB, Hardy Diagnostics, Santa Maria, CA, United States) at 37°C in a shaking incubator (VWR, Radnor, PA, United States) for a total of 6 h to capture bacterial growth at logarithmic phase. Prior to generation and collection of supernatants, growth curves were assessed for all strains to determine the growth cycle of each strain.

Minimum inhibitory concentrations (MIC) were determined using *E*-test (bioMerieux, Marcy-l′Étoile, France) and microbroth dilution assay. Antibiotics were added following 2 h of growth at concentrations that achieve optimal pharmacodynamic parameter for efficacy: vancomycin (unbound) exposure at area under the curve over 24 h (AUC_24_)/MIC 200; ceftaroline at 5× MIC; and tedizolid at AUC_24_/MIC 20. Clinical formulations of vancomycin (Hospira, Inc., Lake Forest, IL, United States) and tedizolid (Merck & Co., Inc., Kenilworth, NJ, United States) were obtained from a hospital clinical pharmacy unit. The active form of ceftaroline was provided by the manufacturer (Allergan, Dublin, Ireland). Supernatants were collected at 6 h and filtered using a 0.45 μm syringe filter (VWR, Radnor, PA, United States) and stored in a −80°C freezer (Thermo Fisher Scientific, Waltham, MA, United States) until use. Bacterial growth was assessed by evaluating colony forming units (CFU) on tryptic soy agar (Hardy Diagnostics, Santa Maria, CA, United States) at 0 h, 2 h, and 6 h. Briefly, bacterial cultures were diluted with phosphate buffered saline (VWR, Radnor, PA, United States) and plated at the −1 through −6 dilutions, with 10 μl added to each TSA plate.

### LTA ELISA

An ELISA specific for detection of *S. aureus* LTA was performed as described in Lotz et al. (2006). Briefly, commercially obtained purified LTA derived from *S. aureus* (InvivoGen, San Diego, CA, United States) was used to establish a standard curve ranging from 31.2 to 2000 ng/ml. In each well, 100 μl of the standard curve or bacterial supernatant samples were incubated in a 96-well Nunc Polysorp plate (Thermo Fisher Scientific, Waltham, MA, United States) for 2 h at room temperature with shaking. After washing three times with 300 μl wash buffer (PBS and 0.05% Tween 20), wells were blocked with 0.5% bovine serum albumin (Sigma-Aldrich, St. Louis, MO, United States) on a shaking incubator (VWR, Radnor, PA, United States) for 1 h. Following blocking, the wells were incubated with 1.2 μg/ml mouse IgG1 anti-Gram-positive LTA antibody (GeneTex, Irvine, CA, United States) at 37°C for 1 h with shaking. The wells were washed three times with wash buffer as stated above and incubated on a shaking incubator at 37°C for 1.5 h with 2 μg/ml goat anti-mouse IgG-HRP conjugated detection antibody (Cell Signaling Technology, Danvers, MA, United States). Again, the wells were washed three times with wash buffer and then incubated with 3,3′,5,5′-Tetramethylbenzidine (TMB) substrate (BioLegend, San Diego, CA, United States) for 10 min in the dark. The reaction was stopped using 1 M H2SO4 stop solution (Millipore Sigma, Burlington, MA, United States) and plate absorbance was measured at 450 nm immediately on a Tecan Sunrise Magellan absorbance microplate reader (Tecan, Männedorf, Switzerland). The assay was repeated with supernatants diluted 1:4 in TSB if concentrations were determined to be above the standard curve. LTA concentration was normalized to CFU count to allow for comparison between strains and antibiotic conditions. The median CFU-adjusted LTA release of the seven clinical strains tested was 7.66e−006 ng. The strain (HH37) associated with the shortest duration of bacteremia and patient survival had the highest CFU-adjusted LTA release (0.00201 ± 0.00039 ng), whereas the two strains (HH70 and HH35) associated with prolonged bacteremia in the blood (11 and 17 days, respectively) and deaths had the lowest release (1.70e−006 ng and 1.81e−006 ng, respectively).

### Whole Genome Sequencing of Bacterial Isolates

#### Bacterial Genomic DNA Extraction

Clinical isolates were streaked and grown on tryptic soy agar overnight prior to harvest for DNA extraction. DNA was extracted using the Qiagen QIAamp DNA Mini Kit (Qiagen, Hilden, Germany) using the manufacturer’s modified protocol for gram positive bacteria, including a mechanical lysis step using the Qiagen TissueLyser. DNA quantification was assessed using Qubit fluorometric quantitation (Thermo Fisher Scientific, Waltham, MA, United States). DNA quality was analyzed using NanoDrop spectrophotometry (Thermo Fisher Scientific, Waltham, MA, United States), wherein absorbance ratios (260/280; 260/230) were used to determine sample impurities.

#### DNA Library Preparation and Whole Genome Sequencing

DNA libraries were prepared for sequencing using the Illumina Nextera XT kit (Illumina, San Diego, CA, United States) as per manufacturer’s instructions. Briefly, bacterial genomic DNA was fragmented and tagged with adapters in a process known as tagmentation. PCR was used to label sample libraries with specific i5 and i7 index codes for identification. The tagged and indexed libraries were then washed three times and purified using ethanol and bead purification with Agencourt AMPure XP beads to remove unused indexes (Beckman Coulter, Indianapolis, IN, United States). Before sequencing, the pooled libraries were normalized to increase the likelihood of equal representation of all sample libraries in the pool, diluted to 20 pM, and denatured. A 1% PhiX library was added to the pooled library as a calibration control. The denatured pooled libraries were loaded into the MiSeq reagent cartridge and sequenced using a paired run setup consisting of 2 × 301 cycles. Following the completion of the sequencing run, FASTQ files were generated using BaseSpace (Illumina, San Diego, CA, United States) and exported for further analysis.

### Bacterial Genome Analysis

#### MLST and *spa* Typing

Multilocus sequence typing (MLST) was performed on the paired sequencing reads using the MLST service provided by an online resource, the Center for Genomic Epidemiology (CGE) bioinformatics server. CGE’s “spaTyper” algorithm was used to assess *spa* typing of isolates, and CGE’s MLST tool was used to assign MLST types. CGE’s bioinformatics workflow assembles paired-end Illumina reads using Velvet v1.0.11, VelvetOptimizer v2.1.7, and variants were identified using SAMtools v0.1.12 mpileup (Li et al., 2009; Larsen et al., 2012). MLST types were further confirmed following alignment to USA 300_FPR3757 and assembly using the Complete Genome Analysis toolset provided by the Pathosystems Resource Integration Center (PATRIC) (Wattam et al., 2017). PATRIC Complete Genome Analysis workflow includes assembly using Unicycler and SPAdes, which are optimized for small genome assembly (Bankevich et al., 2012; Wick et al., 2017). The workflow also includes genome annotation using the RAST tool kit (RASTtk). LTA release at baseline and upon exposure to antibiotics were analyzed with respect to ST of the study strains. Table 2 presents an overview of microbial genome statistics, including genome size, GC content, and number of contigs generated from paired sequencing reads.

**TABLE 2:** Overview of microbial genome sequencing and assembly statistics.

**Strain ID**	**GenBank accession ID**	**Genome size (bp)**	**Number of contigs**	**GC content (%)**	**Number of coding sequences (CDS)**
HH35	GCA_014493615.1	2,657,118	139	32.81	2,609
LA82	GCA_014773325.1	2,852,101	174	32.76	2,862
HH70	GCA_014493625.1	2,617,179	108	32.75	2,527
HH92	GCA_014773345.1	2,737,623	314	33.15	2,753
HH131	GCA_014529735.1	2,863,912	134	32.68	2,921
LA164	GCA_014529875.1	2,688,330	61	32.76	2,616
HH37	GCA_014529825.1	2,645,799	280	33.14	2,632

*Bacterial genomes were sequenced using Illumina MiSeq. Paired-end reads were assembled and annotated using PATRIC’s Complete Genome Analysis toolset. Genome assembly accession number IDs were provided via GenBank. The sequence data and annotations for all strains are publicly available under NCBI BioProject PRJNA660878.*

#### Analysis of Nucleotide and Amino Acid Variations

Comparison of strain nucleotide variation was conducted using PATRIC’s Variation Analysis toolset. The workflow included aligning paired end sequencing reads to USA 300_FPR3757 using BWA-mem and variations were assessed using FreeBayes SNP caller (Garrison and Marth, 2012; Li, 2013). While many variations were found, those relating to the LTA biosynthesis pathway were specifically examined. Variations of interest were re-validated using Geneious Prime (Biomatters, Ltd., Auckland, New Zealand) sequencing analysis software by mapping trimmed, error corrected, and normalized contigs to USA 300_FPR3757 and manually searching for single nucleotide polymorphisms (SNP) in genes of interest. An online bioinformatics tool and predictive algorithm, Protein Variation Effect Analyzer (PROVEAN), was used to predict the impact of amino acid substitutions on protein function (Choi et al., 2012). A PROVEAN score of below −2.5 predicted that a given amino acid substitution would have unspecified deleterious effects on protein function. PATRIC was also used to generate an unrooted phylogenetic tree to compare strain relationships and genetic similarities. The tree-building algorithm utilizes MAFFT to align protein and nucleotide sequences, which are then concatenated and assessed using RAxML rapid bootstrapping (Stamatakis, 2014). Genomes from each strain were compared across 100 single-copy genes, consisting of 40,529 aligned amino acids and 121,587 aligned nucleotides. The genes were then assigned mean squared frequency scores to measure variability (scores closer to 1.0 are conserved across strains, whereas scores closer to 0 are highly variable).

### Statistical Analysis

Statistical analysis was performed using GraphPad Prism version 8.0 (GraphPad Software, San Diego, CA, United States) Data are represented through mean and standard error. One-way ANOVA or paired *t*-tests, where applicable, were utilized to assess statistical differences between treatment groups. *P* values ≤0.05 were considered significant.

## Results

### Low LTA Release From *S. aureus* Strains Relates to Persistent Bacteremia

**FIGURE 1:**
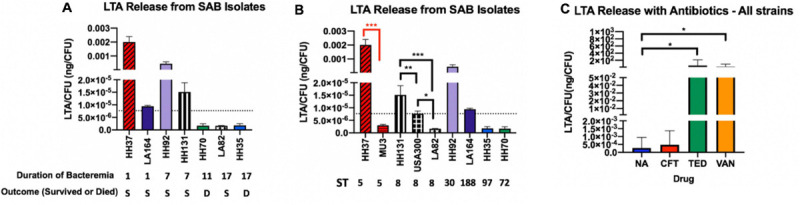
LTA Release from *S. aureus* Bacteremia (SAB) Isolates. **(A)** Strains are grouped based on duration of bacteremia from shortest to longest. **(B)** Strains are grouped by MLST sequence type from highest to lowest release. LTA release was quantified from bacterial supernatants using ELISA. The dotted line in **(A,B)** represents the mean LTA release of all included strains. **(C)** Antibiotics tested include ceftaroline (CFT), tedizolid (TED), and vancomycin (VAN). Antibiotic-treated supernatants were compared with the no antibiotic control (NA). Mean LTA release with antibiotics shows maximal induction with vancomycin and tedizolid. Values are normalized by CFU to control for differences in growth rate between strains. Supernatants were collected from 6-h bacterial cultures and represent the logarithmic phase of growth. Colony forming units (CFU) were determined by plating on tryptic soy agar. Statistical analysis was performed using Student’s *t* test to compare strains of the same sequence type (black brackets, ST8; red brackets, ST5). *p = 0.05; **p = 0.01; ***p = 0.001.

Release of LTA from bacterial cells into the culture supernatant was assessed using ELISA to examine differences in LTA-releasing capabilities between strains that may correlate with clinical phenotypes. The amount of LTA release from seven bloodstream isolates during logarithmic growth phase varied by 4-fold (Figures 1A,B). We assessed the relationship between strain lineage background and LTA release by comparing the amount of LTA release based on MLST sequence type (ST) (Figure 1B). A total of six unique MLST types were represented in our collection of study strains (ST5, ST8, ST97, ST30, ST72, ST188). Notably, LTA release at baseline did not correlate with strain sequence type and differed greatly between strains from the same lineage (ST8 strains: HH131, LA82, and USA 300; ST5 strains: Mu3 and HH37). Interestingly, we observed an association between low LTA release and prolonged duration of bacteremia with or without death. We hypothesized that low LTA release may aid in bacterial immune evasion and prevent the host from mounting a sufficiently robust pro-inflammatory response to facilitate bacterial clearance; this notion would need to be tested in an experimental model of bacteremia.

### *S. aureus* LTA Release in Response to Antibiotic Exposure Was Sequence Type-Specific

In addition to analyzing baseline LTA release from clinical strains (Figure 1), we also examined LTA release in response to antibiotics for the study strains. The agents tested are anti-staphylococcal antibiotics from different pharmacologic classes prescribed in the clinical setting. Vancomycin, a standard treatment for *S. aureus* bacteremia, is a glycopeptide antibiotic that disrupts cell wall synthesis. Ceftaroline, a cephalosporin, also acts on the cell wall, whereas tedizolid is an oxazolidinone and protein synthesis inhibitor that acts on the 50S bacterial ribosome. In general, all antibiotics induced LTA release across strains but to varying degrees. Both tedizolid and vancomycin robustly increased LTA release while ceftaroline contributed only marginally to the increased release of LTA.

Furthermore, we analyzed whether strain response to antibiotic exposure on LTA release was ST-specific. There was a notable trend toward ST8 strains responding differently to antibiotics in comparison to ST5 and other (ST30, ST72, ST97, and ST188) strains, though the differences were not statistically significant due to the relatively small sample size of each sequence type. ST8 strains released maximal LTA in the presence of tedizolid while non-ST8 strains (ST5 and other ST) released maximal LTA in response to vancomycin.

### Genetic Variations in *S. aureus* Bloodstream (SAB) Isolates

USA300_ FPR3757 was selected as the reference strain because of its ubiquity throughout the United States and its strain type, ST8, which is among the most common and was shared between two out of seven clinical strains selected in this study. To assess differences amongst our clinical study strains, we compared SNP-level changes in each strain to determine genetic similarity to USA 300_FPR3757. We identified missense mutations in DNA sequences relative to USA 300_FPR3757 that impact the amino acid sequence of the protein, which may affect the protein’s function by causing misfolding, loss of function, or other errors. The ST8 clinical strains contained the fewest number of mutations overall, due to their relatedness and similarity to USA 300 which also originated from the ST8 lineage. The ST5 strain, HH37, contained the highest number of missense mutations and genes with loss of start or stop codons.

**FIGURE 2:**
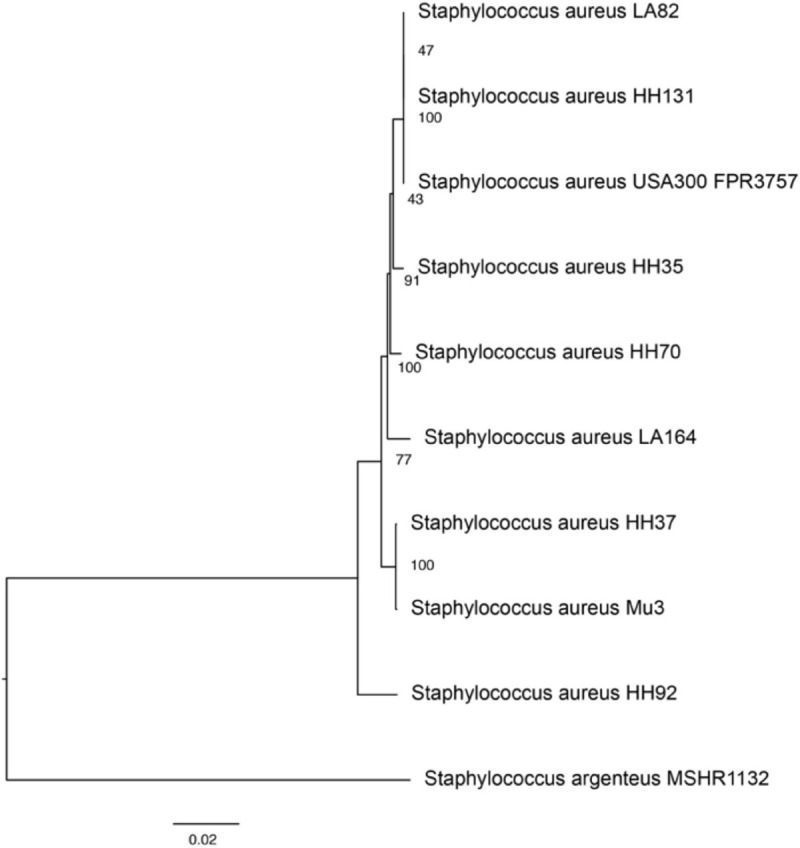
Phylogenetic tree demonstrating genetic similarities between study and reference strains. The phylogenetic tree was generated using PATRIC’s phylogenetic tree algorithm and toolset, which utilizes MAFFT and RAxML rapid bootstrapping to assess relationships between strains. Bootstrapping confidence intervals, depicting the statistical likelihood of the relationship between strains, are displayed between branches. *Staphylococcus argenteus MSHR1132 was* utilized as an outgroup strain to construct the phylogenetic tree.

A phylogenetic tree depicting strain relatedness was generated to compare taxonomy and genetic similarity across strains (Figure 2). *Staphylococcus argenteus MSHR1132*, a closely related species, was utilized as an outgroup strain to define the root of the tree. The phylogenetic tree and accompanying bootstrapping confidence levels support the observation that the ST8 strains HH131, LA82, and USA 300 are closely related and are in the same phylogenetic group; the three ST8 strains form a larger and more divergent group with HH35 and HH70. The ST5 strains HH37 and Mu3 are closely related and comprise a separate, distinct phylogenetic group. LA164 and HH92 are in unique phylogenetic groups and are not clearly related to the other strains or to each other. Among 100 single-copy genes compared across strains, the highest sequence diversity was observed in the gene encoding SarR, which is a protein that participates in the regulatory networks of virulence factor expression, with a mean squared frequency score of 0.630. The least variable gene across strains encodes for the recombination inhibitory protein MutS2, an endonuclease which suppresses homologous recombination during DNA repair and has been shown to limit recombination events and bacterial genetic diversity across strains (Pinto et al., 2005). MutS2 had a mean squared frequency score of 0.997, and was closely followed by guanosine-3′,5′-bis(diphosphate) 3′-pyrophosphohydrolase, a transferase involved in purine metabolism, with a mean squared frequency score of 0.995.

### Genetic Variations Within the LTA Biosynthesis, Modification, and Export Pathways

To better characterize the observed differential release of LTA across clinical strains, we explored genetic variations in the LTA biosynthesis pathway that may lead to increased or decreased release and/or synthesis of LTA. While there were many mutations that differentiated strains outside of ST8 from the USA 300 reference genome, the observed differential LTA release demonstrated across strains did not appear to be associated with ST. We analyzed the genomes of each strain to assess mutations in LTA biosynthetic genes that could potentially effect strain-specific differences in LTA release. Specific genes of interest included: lipoteichoic acid synthase (*ltaS)*; D-alanine–poly(phosphoribitol) ligase subunit 1 (*dltA)*; Poly(glycerophosphate chain) D-alanine transfer protein (*dltD)*; Processive diacylglycerol beta-glucosyltransferase (*ypfP)*; and *SAUSA300_0134* (*rfbX*), also known as polysaccharide extrusion protein, a relatively poorly characterized membrane protein involved in LTA export (Kiriukhin et al., 2001; Lu et al., 2009).

All of the strains, outside of ST8, demonstrated mutations within the LTA biosynthesis pathway relative to the reference strain, with the highest number of unique mutations across genes found in HH92, a high-releasing ST30 persistent strain. Overall the high releasing strains (HH37, HH92) had mutations in: the *ltaS* intergenic region, *dltA*, *dltD*, *rfxB*, and the *rfxB* intergenic regions. The mutations found in the high-releasing strains were unique, as these strains did not share any of the same mutations in the *ltaS* intergenic region or *dltA*. On the other hand, the strains shared several amino acid mutations in *rfxB* which included: Ile360Met and Ile370Leu and Ile371Val. There were no mutations shared by all of the non-ST8 persistent strains (HH37, HH70, HH92); a similar finding is observed for the resolving strains (LA164, HH37), which share none of the same genetic variations.

The LTA biosynthesis-related mutations observed were assessed using PROVEAN scores to predict whether the mutations would have potential impact upon protein biological function. Of the 34 mutations found across strains, the majority were not predicted to be deleterious (Supplementary Table 1). A mutation in *dltA* in strain HH37 (Pro318Leu, PROVEAN score −5.293) and a mutation in *rfxB* in strain HH92 (Ala127Glu, PROVEAN score −3.230) were predicted to be deleterious, but additional information is needed to assess their relative impact upon LTA release as well as the wider prevalence of these mutations amongst clinical *S. aureus* isolates.

When sequencing reads were aligned to USA 300, the ST8 strains LA82 and HH131 had no variations related in the selected LTA biosynthesis genes or intergenic regions, suggesting that the LTA biosynthesis pathway may be conserved across ST8 strains, though additional strains are needed to confirm this finding. The genetic differences in these strains that confer differential LTA-releasing capabilities may be attributed to factors outside of the LTA pathway and/or features of LTA biosynthesis that have not yet been identified and characterized. Additionally, post-translational modifications and changes in mRNA production, as well as changes in transcriptional regulators and non-coding RNA regulation, could also affect differential LTA release.

## Discussion

In this study, bloodstream isolates of *S. aureus* were examined for their LTA-releasing capability with or without the presence of antibiotics in association with varied clinical phenotypes. While detailed investigations were performed on a limited set of study strains, those strains collectively represented six different strain backgrounds (MLSTs), which allowed us to examine strain-specific differences in LTA release in the presence or absence of antibiotics. We observed 4-fold differences in LTA release across strains causing bloodstream infections with distinct clinical presentation and patient outcomes. The observed trend of lower LTA release with more prolonged bacterial growth in blood of patients with *S. aureus* bacteremia deserves confirmation with additional patients and bacterial isolates.

Antibiotic effects on LTA release have been previously reported wherein flucloxacillin, and ciprofloxacin were shown to induce LTA release while the protein synthesis inhibitors, erythromycin and clindamycin, exerted inhibitory effects (van Langevelde et al., 1998; Lotz et al., 2004). Vancomycin has not been previously studied, however, its antimicrobial action on the cell wall may play a role in enhancing LTA release. Another anti-staphylococcal antibiotic not previously studied for its effects on LTA, tedizolid, an oxazolidinone protein synthesis inhibitor, was shown here to increase LTA release (Lotz et al., 2006). This result was unexpected as other studies have reported suppression of LTA release with protein synthesis inhibitors in laboratory strains of *S. aureus*. It is possible that tedizolid could affect strains differently due to unique differences in their membranes or cell walls. While some of the strains selected were shown to be closely related (HH131, LA82), through ST type as well as phylogenetic analysis, others exhibited greater divergence when compared to each other (HH92, LA164). The high level of divergence across clinical *S. aureus* strains causing bloodstream infection likely contributed to the observed heterogeneity in LTA release in the presence and absence of antibiotics and patient outcomes. In this study, across strain genomes, the most genetic heterogeneity was observed in *sarR* which encodes for a repressor of the accessory gene regulator (*agr*), thereby modulating virulence factor expression (Reyes et al., 2011). The relationship between SarR, *agr*, and LTA is currently unknown, though it has been shown previously that wall teichoic acid synthesis is inhibited by enhanced *agr* function. While our work and others have shown that LTA is a key virulence factor that modulates the host immune response, additional data is needed to further explore the functional relationship between teichoic and lipoteichoic acids and overall strain virulence.

Similarly, differences in antibiotic-induced LTA release across clinical strains may be related to changes in cell wall structure conferred by antibiotic resistance mechanisms. LTA biosynthesis is a highly conserved function in Gram-positive organisms. Point mutations causing amino acid variants can possibly alter protein production, protein-protein interactions, protein structure, and/or protein function (McLean et al., 2019). Previous studies have similarly identified genetic differences in LTA biosynthesis leading to structural changes in *Streptococcus suis* strains, which may have implications for influencing the host immune response, though this has not been extensively studied in *S. aureus* clinical strains (Gisch et al., 2018). In this study, we identified genetic differences in LTA biosynthesis and modification pathways in clinical strains causing bloodstream infection, including variants in *rfbX* and *dltA*, in addition to several point mutations in *ygfP*, *ltaS*, and *dltD* of unknown significance. No previously published studies have examined specific mutations in *S. aureus* LTA biosynthesis pathways that correspond with differential LTA release from the cell wall. Further experiments to confirm the contribution of these mutations to the observed differential LTA release across strains using whole transcriptomic approach to assess LTA-related gene expression in clinical strains and changes in gene expression level as the infection evolves would be of interest.

Taken together, the findings of heterogeneity in LTA release across clinical *S. aureus* bloodstream isolates with known clinical phenotypes and the strain background-specific response in LTA release in the presence of antibiotics have potential therapeutic implications and deserve confirmation with larger number of strains with known clinical phenotypes. Additional studies are needed to better understand the clinical relevance of the identified genetic variations and the numerous potential mechanisms leading to differential LTA release and their interactions with antibiotics. Future investigations should examine the relationship between LTA release-mediated host immune response and clinical outcomes as well as the potential for antibiotic modulation of LTA release as a therapeutic strategy to drive LTA-mediated host immune response toward a favorable outcome.

## Data Availability Statement

The datasets presented in this study can be found in online repositories. The names of the repository/repositories and accession number(s) can be found below: https://www.ncbi.nlm.nih.gov/genbank/, JACUZK000000000; https://www.ncbi.nlm.nih.gov/genbank/, JACUZJ000000000; https://www.ncbi.nlm.nih.gov/genbank/, JACVFF000000000; https://www.ncbi.nlm.nih.gov/genbank/, JACVFE000000000; https://www.ncbi.nlm.nih.gov/genbank/, JACVFD000000000; https://www.ncbi.nlm.nih.gov/genbank/, JACWUM000000000; https://www.ncbi.nlm.nih.gov/genbank/, JACWUL000000000.

## Ethics Statement

The studies involving human participants were reviewed and approved by the Institutional Review Boards of University of Southern California and Huntington Hospital. Written informed consent for participation was not required for this study in accordance with the national legislation and the institutional requirements.

## Author Contributions

AW-B and MA conceived the project and designed the experimental plan. MA conducted the experiments and performed the data analyses with support from PJ and AW-B. AW-B, PJ, and MA authored and edited the manuscript. All authors contributed to the article and approved the submitted version.

## Conflict of Interest

Forest Laboratories, now Allergan, which provided partial funding for this study, had no role in study design, data collection and interpretation, or the decision to submit the work for publication.
