# Supplementary material for: Variable Release of Lipoteichoic Acid From Staphylococcus aureus Bloodstream Isolates Relates to Distinct Clinical Phenotypes, Strain Background, and Antibiotic Exposure
Source: Front Microbiol. 2021 Jan 14;11:609280. doi: 10.3389/fmicb.2020.609280 (PMC7840697; doi:10.3389/fmicb.2020.609280)
Supplement: Supplementary file 1 [file Table_1.DOCX]

Supplementary Material

# TITLE: Variable Release of Lipoteichoic Acid from S. aureus Bloodstream Isolates Relates to Distinct Clinical Phenotypes, Strain Background, and Antibiotic Exposure

# AUTHORS & AFFILIATION: Marquerita Algorri^1^, Peter Jorth^2^, and Annie Wong-Beringer^1*^

# 1. University of Southern California, School of Pharmacy, Los Angeles, California, United States; 2. Departments of Pathology and Laboratory Medicine, Medicine, and Biomedical Sciences, Cedars-Sinai Medical Center, Los Angeles, CA 90048, USA

# Supplementary Data

***Table S1.*** *Whole genome data was obtained from SAB isolates using the Illumina Nextera XT DNA Library kit and the Illumina MiSeq sequencing platform. Bacterial genomes and protein sequences were analyzed for variations using PATRIC bioinformatics tools to align, assemble, and compare strains to USA300_FPR3757. PROVEAN was used to determine the predicted impact of single point mutations on protein function.*

**PROVEAN is unable to predict multiple amino acid mutation impact for bacterial species. Nucleic acid variances leading to more than one amino acid substitution have not been assessed. Nucleotide variances found in intergenic regions are nonprotein coding and thus do not correspond directly to amino acid changes.*

| **Gene** | **Gene Product** | **Nucleotide Change (USA300🡪ISOLATE)** | **Amino Acid Change** | **PROVEAN SCORE** | **Strains Affected** |
| --- | --- | --- | --- | --- | --- |
| *ltaS* | Lipoteichoic acid synthase | ATT 🡪 CTT | Ile129Leu | 0.083 | HH35 |
|  |  | CAA 🡪 AAA | Gln233Lys | -0.888 | LA164 |
| *ltaS* (Intergenic region) | N/A | C 🡪 T | -- | -- | HH35, HH70 |
|  |  | G 🡪 A | -- | -- | HH35, HH92 |
|  |  | GTTTTTATTATG 🡪 GTTTTATTATG | -- | -- | HH35, HH70 |
|  |  | A 🡪 T | -- | -- | HH70 |
|  |  | T 🡪 A | -- | -- | LA164 |
|  |  | A 🡪 G | -- | -- | LA164 |
| *dltA* | D-alanine--poly(phosphoribitol) ligase subunit 1 | GAA 🡪 AAA | Gly177Lys | 0.044 | HH37 |
|  |  | GTAAAC 🡪 GTGAG | ValAsn274ValSer | * | HH37 |
|  |  | CCA 🡪 CTA | Pro318Leu | **-5.293** | HH37 |
|  |  | GAA 🡪 GAC | Glu327Asp | -1.418 | HH37, HH70 |
|  |  | GAAGGC 🡪 GCAGGT | GluGly111AlaGly | * | HH92 |
|  |  | AAC 🡪 AGC | Asn275Ser | -0.400 | HH92 |
| *dltD* | Poly(glycerophosphate chain) D-alanine transfer protein DltD | GAA 🡪 GAT | Glu350Asp | -0.871 | HH35, HH37, HH70, HH92, LA164 |
|  |  | GAC 🡪 GAT | Glu233Asp | 0.536 | HH70 |
| *ypfP* | Processive diacylglycerol beta-glucosyltransferase (LTA membrane anchor) | ACA 🡪 ATA | Thr249Ile | -1.355 | HH35, HH37, HH70, LA164 |
|  |  | CGA 🡪 CAA | Arg363Gln | 0.546 | HH92 |
| *SAUSA300_0134* / *rfbX* | Membrane protein involved in the export of O-antigen, teichoic acid lipoteichoic acids | ATC 🡪 GTC | Ile334Val | -0.492 | HH35 |
|  |  | TTC 🡪 ATC | Phe392Ile | 0.469 | HH35, HH37, HH70, LA164 |
|  |  | ATT 🡪 GTT | Ile399Val | -0.055 | HH35, HH37, HH70, LA164 |
|  |  | GCAGTGATAAACCTA 🡪 GCGGTAATGAATTTA | AlaValIleAsnLeu  363AlaValMetAsn  Leu | * | HH37, HH70, HH92 |
|  |  | ATTATT 🡪 CTTGTT | IleIle371LeuVal | * | HH37, HHH70, HH92 |
|  |  | CAT 🡪 CTT | His470Leu | 1.791 | HH70 |
|  |  | GCA 🡪 GAA | Ala127Glu | **-3.230** | HH92 |
|  |  | GGT 🡪 GTT | Gly152Val | 5.004 | HH92 |
|  |  | TCT 🡪 TGT | Ser273Cys | -0.885 | HH92 |
|  |  | ATG 🡪 TTG | Met457Leu | 0.466 | LA164 |
|  |  | C🡪 T | -- | -- | HH35, HH37, HH92, LA164 |
| *SAUSA300_0134* Intergenic region | N/A  Potential regulatory role | T 🡪 C | -- | -- | HH37, HH92, LA164 |
|  |  | A 🡪 T | -- | -- | HH37, HH70, HH92, LA164 |
|  |  | T 🡪 A | -- | -- | HH92 |
|  |  | T 🡪 G | -- | -- | LA164 |
|  |  | C 🡪 T | -- | -- | LA164 |
